# Cytogenetic Abnormalities in Pediatric Myelodysplastic Syndrome: Insights on the Disease Biology and Impact on Leukemic Evolution

**DOI:** 10.3390/biomedicines13122923

**Published:** 2025-11-28

**Authors:** Beatriz Ferreira da Silva, Viviane Lamim Lovatel, Gabriela Farias Lima, Giulia Miceli Giglio Rodrigues, Maria Luiza Rocha da Rosa Borges, Rita de Cássia Barbosa Tavares, Amanda Suhett Fonte, Patricia Regina Cavalcanti Barbosa Horn, Marilza de Moura Ribeiro-Carvalho, Maria Helena Faria Ornellas de Souza, Ana Paula Silva Bueno, Elaine Sobral Costa, Terezinha de Jesus Salles, Teresa de Souza Fernandez

**Affiliations:** 1 Cytogenetic Laboratory, Cell and Gene Therapy Program, Instituto Nacional de Câncer (INCA), Rio de Janeiro 20230-130, RJ, Brazil; 2 Pediatric Oncohematology Center, Oswaldo Cruz University Hospital, Recife 50100-130, PE, Brazil; 3 Bone Marrow Transplantation Center (CEMO), Instituto Nacional de Câncer (INCA), Rio de Janeiro 20230-130, RJ, Brazil; 4 Pediatric Hematology Department, Hospital Federal da Lagoa, Rio de Janeiro 22470-050, RJ, Brazil; 5 Departament of General Pathology, Faculty of Medical Sciences, Universidade Estadual do Rio de Janeiro (UERJ), Rio de Janeiro 20550-170, RJ, Brazil; 6 Faculty of Medicine, Pediatric and Puericulture Martagão Gesteira Institute (IPPMG), Universidade Federal do Rio de Janeiro (UFRJ), Rio de Janeiro 21941-912, RJ, Brazil

**Keywords:** Pediatric MDS, cytogenetic alterations, leukemic evolution

## Abstract

**Background/Objectives:** Pediatric myelodysplastic syndrome (pMDS) is a rare, heterogeneous, clonal hematopoietic stem cell disease with a risk of evolution to acute myeloid leukemia (AML). Clonal cytogenetic abnormalities are present in 30–60% of pMDS. However, unlike in adults, the prognostic significance of chromosomal alterations, particularly their role in predicting evolution to AML, remains limited in pMDS. To acknowledge this gap, we studied the cytogenetic abnormalities in pMDS and analyzed their associations with subtypes and evolution to AML. Furthermore, in the Discussion Section, we pointed out key genes involved in these chromosomal alterations. **Methods:** Cytogenetic analysis was performed on 193 pediatric patients using G-banding and fluorescence in situ hybridization. **Results:** Abnormal karyotypes were observed in 43.5% (84/193) of patients, mainly in the advanced subtype. The main chromosomal alterations were monosomy 7 (−7) in 14% of the cases (12/84), deletion of the long arm of chromosome 11 [del(11q)] in 12% (10/84) and both trisomy 8 (+8) and deletion of the long arm of chromosome 7 [del(7q)] in 8% (7/84). Evolution from MDS to AML was observed in 22% of the patients (42/193), and it was associated with complex karyotypes, del(11q), −7/del(7q), and +8. **Conclusions:** Our results suggest that specific chromosomal alterations, such as del(11q), del(7q), and +8, may predict evolution to AML and might be considered high-risk cytogenetic markers in pMDS.

## 1. Introduction

Myelodysplastic syndrome (MDS) comprises a heterogeneous group of hematopoietic stem cell clonal neoplasms. Dysplastic alterations in the bone marrow (BM), ineffective hematopoiesis, and variable degrees of cytopenias in the peripheral blood are features observed in MDS [1]. In the pediatric age group, MDS (pMDS) is a rare disease, representing less than 5% of all hematopoietic neoplasms of childhood [1,2]. Pediatric patients with MDS show genetic characteristics different from those observed in adult patients, which reflects in differences in their clinicopathology [1,2,3]. Beyond that, patients with pMDS usually have a high risk of evolution to acute myeloid leukemia (AML) [1,2,3,4].

Clonal cytogenetic alterations are usually present in approximately 30–60% of pMDS patients, mainly in advanced subtypes [5,6,7]. Cytogenetic alterations have contributed to the identification of mechanisms involved in disease biology and the development of treatments [1,4]. The cytogenetic hallmarks of MDS are partial or total chromosomal losses (deletions or monosomies) and chromosomal gains (trisomies) [8]. These recurrent chromosomal alterations indicate the presence of tumor suppressor genes and oncogenes, which contribute to our understanding of the disease biology and its clinical course [9]. Monosomy 7 (−7) is the most common cytogenetic alteration in pMDS, occurring in approximately 30% of the cases, followed by trisomy 8 (+8), trisomy 21 (+21), and complex karyotypes [5,10,11].

Cytogenetic analysis is an important variable in prognostic risk stratification, being incorporated in the International Prognostic Scoring System (IPSS) and later in the revised IPSS (IPSS-R) [12,13,14,15]. The cytogenetic prognostic risk according to IPSS-R recognizes five groups: very good, good, intermediate, poor, and very poor. Monosomy 7 is associated with evolution from MDS to AML and a poor prognosis [15,16,17]. This chromosomal abnormality has been widely studied at the molecular level and has indicated the relevance of genes, such as *SAMD9/SAMD9L* and *EZH2*, for disease pathogenesis [10,11,16,18,19]. However, the association between other specific chromosomal abnormalities and leukemic evolution in pMDS has been rarely reported [16,17,20]. And also, the genes involved in these chromosomal alterations are not commonly discussed. Therefore, the aim of this study was to analyze the frequency of cytogenetic alterations in pMDS, their association with pMDS subtypes and evolution to AML, highlighting the key genes implicated in the main cytogenetic abnormalities in pMDS.

## 2. Material and Methods

### 2.1. Patients

Bone marrow (BM) cells were obtained from 193 pediatric patients with MDS between 2000 and 2023. Chromosomal and clinical studies were carried out in all cases. Patients were diagnosed at the Instituto Nacional de Câncer (INCA, Rio de Janeiro), Instituto de Pediatria e Puericultura Martagão Gesteira (IPPMG, UFRJ), Hospital Universitário Pedro Ernesto (HUPE, Rio de Janeiro), and Hospital Universitário Oswaldo Cruz (Recife, Pernambuco). Among the 193 patients, there were 114 males and 79 females; the mean age was 9 years, ranging from 3 months to 18 years old. None of these patients had been treated for malignancy previously, nor did they have a previous diagnosis of genetic syndromes. The diagnosis was performed according to clinical, morphological, immunophenotypic, and cytogenetic characteristics. The pMDS classification was performed according to the International Consensus Classification (ICC) [4]. The clinical characteristics of the patients are described in Table 1.

### 2.2. Conventional and Molecular Cytogenetic Analysis

The BM aspirates were collected in heparinized tubes. Cells (5 × 10^6^) were cultured in RPMI 1640 medium supplemented with 20% fetal calf serum at 37 °C, 5% CO_2_ for 24 h. In the final hour of incubation, the cultures were pulsed with colcemid (0.05 µg/mL). Following incubation, cells were harvested by standard procedures (hypotonic shock: 0.075M) and fixed in methanol/acetic acid (3:1). The chromosomal analyses were performed by G-banding using 10 slides per patient as previously described [14]. The fixed cells were dropped onto clean slides, flame-fixed with a Bunsen burner, and aged for 24 h at room temperature. Slides were treated with 0.1% trypsin in Dulbecco’s solution (8 g NaCl, 0.2 g KCl, 0.2 g KH_2_PO_4_, 1.5 g NaH_2_PO_4_ per 1 L distilled water, pH 7.8) for 1 s to 1 min, then rinsed immediately in 0.9% NaCl. The slides were stained in 2% Giemsa in phosphate buffer (14.075 g NaH_2_PO_4_ per 1 L distilled water, pH 6.8) for 15 min. Chromosomes were classified according to ISCN 2020 [21], and karyotype images were acquired using the Ikaros Karyotyping System (MetaSystems, Zeiss, Altlussheim, Germany).

To confirm chromosomal alterations or to characterize the breakpoint and the gene involved in chromosomal abnormality, fluorescence in situ hybridization (FISH) analyses were performed. Slides were prepared from cytogenetic cultures and dried on a heated plate at 42 °C. The slides were incubated in 2× SSC (20× SSC: 3.0 M NaCl, 0.3 M sodium citrate, pH 7.0) for 20 min at room temperature, then in ethanol (70%, 90%, 100%, 2 min each). FISH analysis was performed using the following probes: −7/del(7q) (D7S486 spectrum orange/CEP7 spectrum green), +8 (LSI cMYC, spectrum orange), del(11)(q23) (LSI MLL dual-color break-apart rearrangement probe), del(17)(p13) (LSI p53, spectrum orange), del(5)(q31) (LSI CSF1R “spectrum orange”/LSID5S23:D5S721 spectrum green) (Vyses, Abbott Laboratories, Des Plaines, IL, USA). Probes were prepared according to the manufacturer’s instructions, covered with a coverslip, and sealed with rubber cement (Marabu, Tamm, Germany). Hybridization was performed at 37 °C for 16 h in a Thermobrite hybridization chamber (Leica, Richmond, VA, USA). Then, the slides were washed in 0.4× SSC + 0.3% Tween at 73 °C for 2 min, followed by 0.2× SSC + 0.1% Tween at room temperature for 1 min. Slides were counterstained with DAPI and analyzed by fluorescence microscopy (Olympus BX51, Olympus Corporation, Maimi, FL, USA). Images were acquired with the ISIS imaging system (MetaSystems, Zeiss, Altlussheim, Germany).

**Figure 1:**
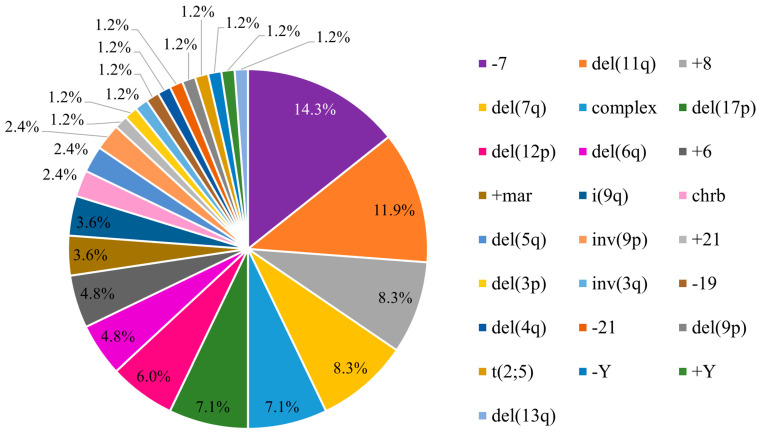
Frequency of clonal chromosomal abnormalities in pMDS.

**Figure 2:**
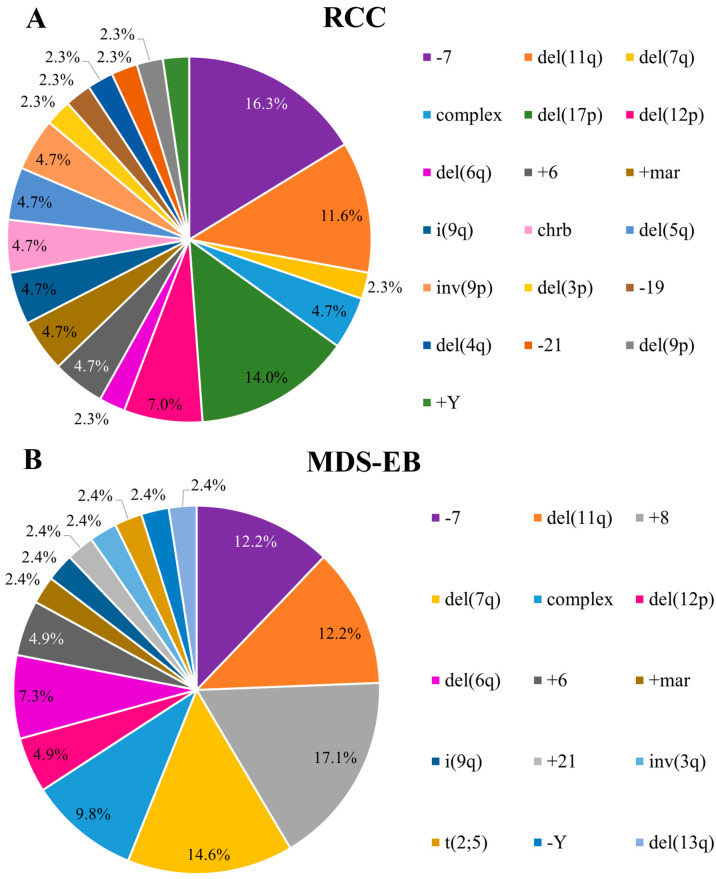
Frequency of clonal chromosomal abnormalities according to pMDS subtypes: (**A**) Refractory Cytopenia of Childhood (RCC); (**B**) MDS with Excess Blasts (MDS-EB).

For the analysis of the frequency of chromosomal alterations (Figure 1 and Figure 2), complex karyotypes (defined as ≥3 abnormalities) were counted as an independent category. In cases with double chromosomal abnormalities, it was considered the alteration associated with the worst prognosis according to the IPSS-R.

The statistical difference between normal and abnormal karyotypes in relation to leukemic evolution was analyzed through a chi-square test. Logistic regression was used to verify the probability of AML evolution occurrence in relation to specific cytogenetic alteration, which calculated the odds ratio (OR), with 95% confidence interval and *p*-value. Statistical analyses were performed with SPSS 20.0 software. The value of *p* < 0.05 was considered statistically significant in all the analyses.

## 3. Results

### 3.1. Cytogenetic Abnormalities in Pediatric Myelodysplastic Syndrome

In the present study, abnormal karyotypes were identified in 43.5% of patients (84/193). Monosomy 7 was the most frequent alteration, detected in 14% (12/84) of cases, while del(7q) occurred in 8% (7/84). The second most common abnormality was the deletion of the long arm of chromosome 11 [del(11q23)] in 12% (10/84), followed by trisomy 8, present in 8% (7/84) of patients, and both complex karyotypes (≥3 abnormalities) and deletion of the short arm of chromosomes 17 [del(17p)] in 7% (6/84). Other chromosomal alterations were observed, such as deletion of the short arm of chromosome 12 [del(12p)], deletion of the long arm of chromosome 6 [del(6q)], and trisomy of chromosome 6 (+6), representing 6%, 5%, and 5%, respectively. The remaining chromosomal alterations had a frequency of less than 4%. The frequency of clonal cytogenetic abnormalities in pMDS is shown in Figure 1.

### 3.2. Distribution of the Chromosomal Pattern of pMDS Patients According to Subtypes

According to the pMDS classification proposed by ICC, 74.6% (144/193) were classified with the initial subtype of the disease, the refractory cytopenia of childhood (RCC), and 25.4% (49/193) with the advanced subtype, MDS with excess blasts (MDS-EB). Abnormal karyotypes were observed in 30% (43/144) of RCC and 83% (41/49) of MDS-EB (Figure 2).

### 3.3. Cytogenetic Risk Groups According to IPSS-R

The cytogenetic risk groups according to IPSS-R showed the following distribution: 5.7% (11/193) were very good; 60.1% (116/193) were good; 23.3% (45/193) were intermediate; 8.3% (16/193) were poor; and only 2.6% (5/193) were very poor. According to this distribution, the disease progression occurred in 64% (7/11) of patients in the cytogenetic group with a very good risk, in 6% (7/116) with a good risk, in 33.3% (15/45) with intermediate risk, in 56% (9/16) of those considered as poor risk and in 80% (4/5) of the very poor risk group (Table 2).

### 3.4. Association Between the Chromosomal Pattern and Evolution from pMDS to AML

The evolution from pMDS to AML occurred in 22% (42/193) of patients, being significantly associated with abnormal karyotypes (*p* < 0.0001). The distribution according to the pMDS subtypes showed that only 10% (14/144) of the RCC patients had MDS evolution, whereas leukemic transformation in occurred in 57% (28/49) of MDS-EB patients. Abnormal karyotypes were observed in 83% (35/42) of these patients. In the univariate analysis, using the group with a normal karyotype as a reference, it was observed that specific chromosomal abnormalities were significantly associated with the progression of myelodysplastic syndrome (MDS) to acute myeloid leukemia (AML). Patients with a complex karyotype showed a markedly increased risk of progression, with an odds ratio (OR) = 39.6 (95% CI: 6.9–226.8; *p* < 0.001). Similarly, deletion of the long arm of chromosome 11 [del(11q)] was strongly associated with progression, with OR = 27.4 (95% CI: 6.7–111.8; *p* < 0.001). Alterations involving chromosome 7, such as monosomy 7 (OR = 13.7; 95% CI: 3.7–50.9; *p* = 0.0003) and 7q deletion (OR = 17.6; 95% CI: 3.6–85.4; *p* = 0.001), were also significantly related to leukemic transformation, as was trisomy 8 (OR = 17.6; 95% CI: 3.6–85.4; *p* = 0.001) (Table 3).

## 4. Discussion

Despite the advancements in genomics, cytogenetic analysis remains essential for MDS diagnosis, prognosis, and clinical decision-making, offering good cost-effectiveness, especially in public hospitals [1,2,3,4]. However, there is a notable gap in the literature on pMDS, particularly regarding cytogenetics in pMDS [5,10,17,22,23,24]. It is important to note that many pMDS studies often include Juvenile Myelomonocytic Leukemia (JMML) and Chronic Myelomonocytic Leukemia (CMML) or secondary pMDS patients, further making it difficult to interpret cytogenetic findings [5,10,17,22,23,25].

In the present study, the cytogenetic analyses were performed in 193 pediatric patients with de novo MDS, focusing mainly in the impact of cytogenetic abnormalities in the evolution from MDS to AML. The overall frequency of abnormal karyotypes in our study was 43.5%, similar to previous studies in pMDS (41–64%) [5,16,22]. Cytogenetic analysis at diagnosis is considered a relevant indicator of leukemic transformation. In addition, a higher frequency of abnormal karyotypes has been described in patients with advanced MDS [5,16,22,26,27]. Corroborating these data, in the present study, patients with MDS-EB had a remarkably higher frequency of abnormal karyotypes than those with RCC, and the evolution from pMDS to AML was significantly associated with abnormal karyotypes (*p* < 0.0001). Regarding specific cytogenetic alterations, in our cohort, complex karyotypes, del(11q) −7/del(7q), and +8 were the most frequent chromosomal alterations associated with the evolution to AML.

Wlodarski and colleagues emphasized that, despite the advent of genomics and transcriptomics, conventional cytogenetics remains the best approach for detecting chromosome 7 abnormalities, particularly clonal evolution, acquisition of new alterations, or identification of independent cytogenetic clones in patients with pMDS [11]. Traditionally, −7 and del(7q) account for the majority of karyotypes in pMDS cases [2,10,27,28]. In the present study, −7 and del(7q) accounted for 23% of abnormal karyotypes, being the most frequent cytogenetic alterations. Its recurrence strongly suggests that the genes in this chromosome have a critical role in disease pathogenesis [9].

For instance, *CUX1*, mapped to 7q22, has been a candidate gene in malignant myeloid disorders with −7/del(7q). *CUX1* is implicated in gene expression regulation, cell differentiation, cell cycle, and DNA repair. This transcription factor is highly expressed in multipotent hematopoietic progenitors and downregulated in −7/del(7q) MDS cases [29,30]. Other genes located in this chromosome, such as *EZH2*, *MLL3*, and *SAMD9/SAMD9L*, have also been associated with MDS pathogenesis [9,11,19,22]. *EZH2* and *MLL3* are histone methyltransferases involved in stem cell regulation and hematopoietic differentiation [19,31]. Our group previously demonstrated that reduced *EZH2* expression is associated with chromosome 7 abnormalities and disease evolution in MDS patients [19]. Furthermore, in vivo studies have shown that the haploinsufficiency of *MLL3* may contribute to the development of myeloid malignancies [31]. Additionally, germline variants in the tumor suppressor genes *SAMD9* and *SAMD9L*, which regulate cell proliferation, are observed in approximately 7% of children who develop MDS with −7/del(7q), usually associated with the initial subtype [31,32].

The heterogeneous nature and rapid clonal expansion of −7/del(7q) represent a challenge to clinical management [9,11,19,23]. Whether del(7q) and −7 have equivalent clinical impact remains unclear, especially in the pediatric age group. Currently, the IPSS-R confers del(7q) an intermediate risk as a single abnormality and a poor risk when observed with an additional chromosomal alteration, while −7 confers a poor risk [15]. Overall, the difference observed in the prognosis of these alterations could reflect the broader loss of chromosomal material and consequential gene dosage in the −7 cases. In our study, 83% of the patients with −7 and 86% of the patients with del(7q) presented them as a sole chromosomal alteration. However, approximately half of the patients with either of these alterations had leukemic evolution. These results might suggest that the loss of tumor suppressor genes in the long arm of chromosome 7 have an important role for the evolution from pMDS to AML.

The +8 is the most common chromosomal gain in pMDS [17,33]. Herein, +8 represented 8% of abnormal karyotypes. Moriwaki and colleagues reported a slightly higher frequency of 16%. However, their cohort also included JMML, CMML, and secondary pMDS patients. Considering only their primary pMDS cases, outside of the context of complex karyotypes, the +8 frequency was similar to our results [7]. Regarding the prognostic impact of this alteration, the IPSS-R classifies this alteration as an intermediate risk despite being observed isolated or accompanied by other abnormalities [15]. However, there are still controversies in the +8 prognostic impact, especially compared to other cytogenetic alterations classified as intermediate [34,35,36]. In our study, +8 was associated with advanced subtypes and disease evolution. 

This observation highlights that the gain in gene dosage associated with this chromosomal alteration may play a pivotal role in the risk of evolution from pMDS to AML. Nevertheless, the +8 pathogenetic mechanism in MDS has not yet been fully elucidated. Among the genes on this chromosome, the main candidate is the *cMYC*. This gene encodes a transcription factor that acts in cell proliferation, differentiation, and maintenance. Its over-expression has been shown to have a central role as a downstream mediator of myeloid neoplasms, promoting both cell proliferation and apoptosis [34,37]. However, the role of *c-MYC* in the pathogenesis of MDS is still poorly understood [34,38].

The IPSS and IPSS-R are the prognostic models most commonly used in MDS. However, these systems were originally developed based on adult patient data and may therefore have limited applicability in pediatric cases [13,15,17]. Yamamoto and colleagues (2021) evaluated the IPSS-R cytogenetic classification in children undergoing hematopoietic stem cell transplantation (HSCT) and demonstrated that, although the very poor-risk IPSS-R cytogenetic category could predict worse outcomes after allogeneic HSCT in pMDS patients, no significant difference was observed between the good and intermediate cytogenetic risk groups. Consequently, they propose that reclassification into three groups (standard, poor, and very poor) instead of the five original IPSS-R classification [17].

It is important to note that, in Yamamoto and colleagues’ study, there were no cases in the very good risk group, which includes patients with -Y and del(11q) [17]. However, in our study, del(11q) was the second most frequent alteration. In the literature, del(11q) is an uncommon clonal abnormality found in 0.6% to 3% of all MDS patients [39], usually associated with other cytogenetic alterations [10,22]. Several genes important for the normal biology of hematopoietic stem cells are located in this chromosome, such as *KMT2A*, *CADM1*, *ATM*, and *CBL* [39]. *KMT2A* plays a role in chromatin remodeling and transcriptional regulation, and its rearrangement is a hallmark of some childhood leukemias [39,40]. Recently, a study investigated the genes involved in del(11q) in MDS and identified that the commonly deleted region primarily affects CADM1. This gene regulates myeloid cell production and terminal differentiation [39].

The very good prognosis status of del(11q) by the IPSS-R was proposed based on the evaluation of 20 patients with this alteration [15,24]. Currently, the largest del(11q) cohort analyzed 103 adult patients and verified that this alteration had a similar prognosis to MDS with other chromosomal alterations [39]. The impact of this alteration in pMDS is still unknown. However, in the present study, the del(11q) was associated with a high frequency of evolution to AML (70%), suggesting that the del(11q) might confer an unfavorable prognosis in pMDS.

In our study, complex karyotypes alongside del(17p) were the fourth most observed cytogenetic pattern (7%). Complex karyotypes are mostly associated with the advanced subtypes and genomic instability that leads to leukemic transformation [20,41]. The complex karyotypes can be classified into two categories: those with both structural and numerical alterations, and those with only numerical changes [6,15]. In our cohort, it was possible to identify patients with only chromosome gains, characterizing a hyperdiploid karyotype. This alteration is not normally reported in MDS. However, the prognostic impact of this type of complex karyotype, alongside other rare chromosomal abnormalities in pMDS, was recently discussed by our group [6]. In this study, complex karyotypes had a higher frequency in advanced subtypes and were associated with disease progression, consistent with their poor/very poor prognosis [15].

In contrast to complex karyotypes, the del(17p), another important alteration involved in leukemogenesis, was associated with the initial subtype, the RCC, and none of our patients with this alteration showed disease progression. It is noteworthy that the 17p13 region harbors the tumor suppressor gene *TP53*, and the biallelic inactivation of the *TP53* gene has been proposed as a distinct subtype in adult patients (MDS-bi*TP53*), given that it leads to loss of function and poor prognosis [1,4,42]. This suggests that the second allele is probably not molecularly altered in our cohort.

Alterations on chromosome 6, such as deletion of the long arm of chromosome 6 (6q), and trisomy 6 (+6) were also observed. Chromosome 6 harbors genes that regulate different aspects of hematopoiesis, such as *HBS1L*, *MYB*, and *AHI1*. The *MYB*, e.g., is a proto-oncogene crucial to hematopoietic development, commitment, and differentiation of cell lineages [43,44]. Therefore, their loss of heterozygosity or amplification can affect hematopoiesis. The loss of heterozygosity of 6q has been commonly described in B or T acute lymphoblastic leukemia (ALL) and chronic lymphocytic leukemia (CLL) [43]. The +6, in turn, has been proposed as a marker of AML patients that results from other myeloid disorders transformation [45]. These alterations are usually associated with additional abnormalities, contrasting with our patients who had del(6q) and +6 as a single alteration. Their prognostic significance is not well established, though the IPSS-R stratifies as an intermediate group [15]. Interestingly, in our study, two patients, one with +6 and one del(6q), classified as MDS-EB had disease evolution to AML as previously observed [46].

In MDS, del(12p) is described in 0.6 to 5% of adult patients at the initial subtype and is stratified as a good prognosis. In the present study, this alteration represented 2.6% of all cases. Alterations between 12p12-p13 can involve the *ETV6* and *CDKN1B* genes, respectively. The *ETV6* gene acts in the establishment of hematopoiesis in all lineages [47]. However, there are no reports about the impact of del(12p) in pMDS. In our cohort, three patients with RCC and two MDS-EB, had del(12p). However, none of them showed disease evolution. 

**Figure 3:**
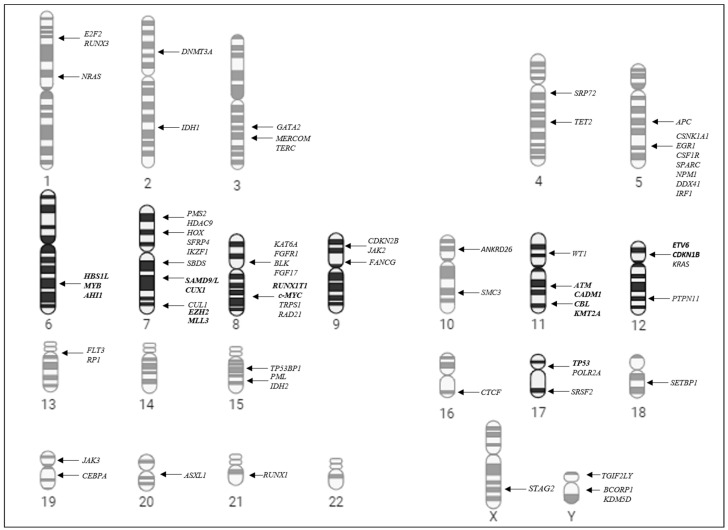
Diagrammatic representation of the karyotype highlighting the main chromosomes involved in pMDS cytogenetic alterations and pointing out important genes.

The study of cytogenetic abnormalities in hematological neoplasms has provided important insights into the molecular mechanisms underlying the pathogenesis of the disease, pointing to important genes [48]. In this context, throughout the discussion of this study, we sought to highlight key genes located in the chromosomal alterations observed in our cohort, which may play a critical role in the biology of pMDS (Figure 3). It is worth mentioning that, although at a lower frequency, leukemic evolution was also observed in patients with a normal karyotype, indicating alterations at the molecular level. This emphasizes that patients with normal karyotypes could benefit from genomic complementary approaches [16,23,49].

In summary, our results suggest that specific chromosomal alterations, such as complex karyotypes, del(11q), −7/del(7q), and +8, may predict evolution to AML. Interestingly, del(11q) is classified as a very good prognosis, and +8 and del(7q) as an intermediate prognosis according to the IPSS-R; however, herein, these alterations showed association with disease evolution to AML. This underscores the need for more pMDS cytogenetic studies that could reflect the distinct cytogenetic risk groups for the pediatric age group.

## 5. Conclusions

This study presents a cytogenetic analysis of a large cohort of pediatric patients with MDS. Abnormal karyotypes were present in 43.5% of patients. Specific chromosomal alterations, such as complex karyotypes, del(11q), −7/del(7q), and +8, were associated with the evolution to AML, underscoring their potential as important prognostic markers in pMDS.

## Figures and Tables

**Table 1:** Clinical characteristics of the 193 pediatric patients with MDS.

Patients	Number (%)
MDS	193
**Gender**	
Male	114 (59%)
Female	79 (41%)
**Mean age (range)**	9 (3 months–18 years)
(0–2 years)	34 (17.6%)
(3–11years)	92 (47.7%)
(12–18 years)	67 (34.7%)
**Number of cytopenias**	
1	57 (29.5%)
2	71 (36.8%)
3	65 (33.7%)
**MDS Subtypes**	
RCC	145 (66%)
MDS-EB	48 (21.8%)
**Cytogenetics**	
Normal	109 (56.5%)
Abnormal	84 (43.5%)
**Evolution from MDS → AML**	
No	151 (78%)
Yes	42 (22%)

RCC: refractory cytopenia of childhood; MDS-EB: MDS with excess blasts.

**Table 2:** Distribution of pMDS patients according to the IPSS-R cytogenetic risk stratification evolution to AML.

Cytogenetic Risk IPSS-R	Frequency %/(Number of Patients)	Evolution MDS to AML
VERY GOOD	5.7% (11/193)	64% (7/11)
GOOD	60.1% (116/193)	6% (7/116)
INTERMEDIATE	23.3% (45/193)	33.3% (15/45)
POOR	8.3% (16/193)	56% (9/16)
VERY POOR	2.6% (5/193)	80% (4/5)

**Table 3:** Association between karyotypes and evolution from pMDS to AML.

Karyotype	% MDS Evolution to AML	Odds Ratio	*p*-Value
normal	6.4% (7/109)		
−7	50% (6/12)	13.67	0.00030
+8	57% (4/7)	17.57	0.00138
del(11q)	70% (7/10)	27.40	<0.0001
complex	83% (5/6)	39.58	<0.0001
del(7q)	57% (4/7)	17.57	0.00138
del(12p)	0% (0/5)	0.06	0.0207
del(17p)	0% (0/6)	0.05	0.0103
del(6q)	25% (1/4)	5.86	0.258
+6	25% (1/4)	5.86	0.258
+mar	0% (0/3)	-	-
chrb	0% (0/2)	-	-
del(5q)	0% (0/2)	-	-
+21	100% (1/1)	-	-
i(9q)	33% (1/3)	-	-
inv(9p)	0% (0/2)	-	-
del(9p)	0% (0/1)	-	-
del(3p)	100% (1/1)	-	-
inv(3q)	0% (0/1)	-	-
−19	0% (0/1)	-	-
del(4q)	100% (1/1)	-	-
−21	100% (1/1)	-	-
t(2;15)	100% (1/1)	-	-
−Y	0% (0/1)	-	-
+Y	0% (0/1)	-	-
del(13q)	100% (1/1)	-	-

Logistic regression was performed for chromosomal alterations that were present in at least four patients.

## Data Availability

The original contributions presented in this study are included in the article. Further inquiries can be directed to the corresponding author.
